# Zoom in on learning with a virtual microscope: A convergent parallel mixed-method study

**DOI:** 10.1371/journal.pone.0323412

**Published:** 2025-05-07

**Authors:** Ava K. Chow, Nazlee Sharmin

**Affiliations:** Mike Petryk School of Dentistry, Faculty of Medicine and Dentistry, College of Health Sciences, University of Alberta,Edmonton, Canada; Bangor University, UNITED KINGDOM OF GREAT BRITAIN AND NORTHERN IRELAND

## Abstract

Knowledge of histology is essential for many disciplines in professional health education. Virtual Microscopes (VMs) are increasingly becoming popular as a cost-effective teaching tool for histology. However, a lack of hands-on experience with traditional light microscopes and glass slides concerns many educators. Although studies have reported improvement or no difference in students’ knowledge and/or performance using either virtual or optical microscopes, reports on the impact of VMs on students’ understanding of magnification and orientation are scarce. We conducted a convergent, parallel mixed-method study to assess dental students’ understanding of magnification and orientation after using a virtual microscope to study oral histology. Six hours of mandatory lab sessions and critical thinking assignment questions were designed for the 1^st^ year students in the Doctor of Dental Surgery (DDS) program. Quantitative data were collected from students’ performance in orientation and magnification-related questions in the summative assessments. Students’ written responses to reflective questions were the qualitative data, analyzed using manifest content analysis. All 32 students accurately answered 3 out of 5 questions, requiring them to apply the knowledge of magnification and orientation. 30 and 29 students correctly answered the remaining questions, respectively. 97% of the class agreed to improve their understanding of magnification and orientation after using the VM. All (100%) students (n = 32) completed the reflective assignment, generating 64 meaning units. 17 codes were generated and compiled into seven subcategories, which were further condensed into two categories: refinement of mental models and enhanced learning. Although our study is limited by a small sample size, it sheds light on the strategies adopted by dental students to improve their senses of magnification and orientation while using a VM.

## Introduction

Knowledge of histology, the study of tissue structures under a microscope, is essential for many disciplines in health professional education. Mazzarini et al. (2021) describe histology as ‘the study of the morphology of the cells in multicellular organisms within their natural environment’ [1]. Traditional ways of teaching histology rely on textbooks, glass slides, and conventional light microscopes. However, over the past decades, virtual microscopes and digital slides have become popular, enabling health professional schools across the world to deliver histology training to a broader audience in a cost-effective manner [2]. The use of virtual microscopes (VMs) is also well-aligned with the strategic goals of the rapidly evolving medical curricula and can address the learning needs of the new generation of students [3].

Virtual microscopy refers to the digital conversion of light microscopic specimens and their presentation over a computer network and display system [4]. The widespread accessibility of VMs is considered to be the key advantage of this technology over light microscopes [5]. Besides ubiquitous availability, VMs positively impact knowledge acquisition, long-term knowledge retention, student performance, and satisfaction [6]. However, similar to any new educational technology, there are some limitations of teaching with VMs, causing reluctance among some educators to embrace this teaching tool. The hands-on skill of operating a microscope is still required for health professionals in some countries [7,8]. Exposure to only high-quality specimens has also been identified as a limitation as it lacks students’ appreciation of variations between slides [9].

Magnification, the apparent size of an object compared to its actual size, is the most essential feature of any microscope [10]. In a traditional light microscope, students manually change the objective lenses and, looking through the eyepiece, focus the magnified image using adjustment knobs. In a virtual microscope, on the other hand, magnification is increased by the click of a mouse without requiring additional steps to adjust the focus of the image, which may make a learner unaware of the scale of magnification of a tissue section. The magnified image also appears different on different display sizes, necessitating the inclusion of a magnification scale in the display system of VMs [10].

The benefit of hands-on learning in science education is attributed to the haptic sensation, which involves touch and intentional movement of objects. When linked with vision, haptic perception helps students understand an object’s shape, structure, texture, and orientation [11]. Understanding the orientation of a slide is also essential to accurately identify a tissue section. Students in a virtual microscopy lab will not access physical glass slides, which may impact their understanding of tissue orientation.

Reports on VMs primarily focus on students’ knowledge of tissue structures, academic performance, ease of navigation, and satisfaction. The impact of VMs, particularly on students’ understanding of magnification and orientation, has been poorly explored. At the Mike Petryk School of Dentistry, we have developed a virtual microscope (VM) called Histoscope (www.histoscope.com/) for teaching oral histology [12]. Histoscope archives a large collection of tissue sections, which includes but are not limited to teeth, gingiva, salivary glands, temporomandibular joint, tongue, palate, developing embryo head, and developing and erupting teeth. Six hours of lab sessions with VM were designed for the 1^st^ year students of the Doctor of Dental Surgery (DDS) program. A study was conducted to assess students’ understanding of magnification and orientation after using Histoscope. Our specific research questions are:

1. How does Histoscope, a VM, impact dental students’ understanding of the magnification and orientation of a tissue section?
2. What are dental students’ perceptions of using a VM to comprehend the magnification and orientation of a tissue section?

## Materials and methods

### Design and implementation of oral histology labs

**Fig 1:**
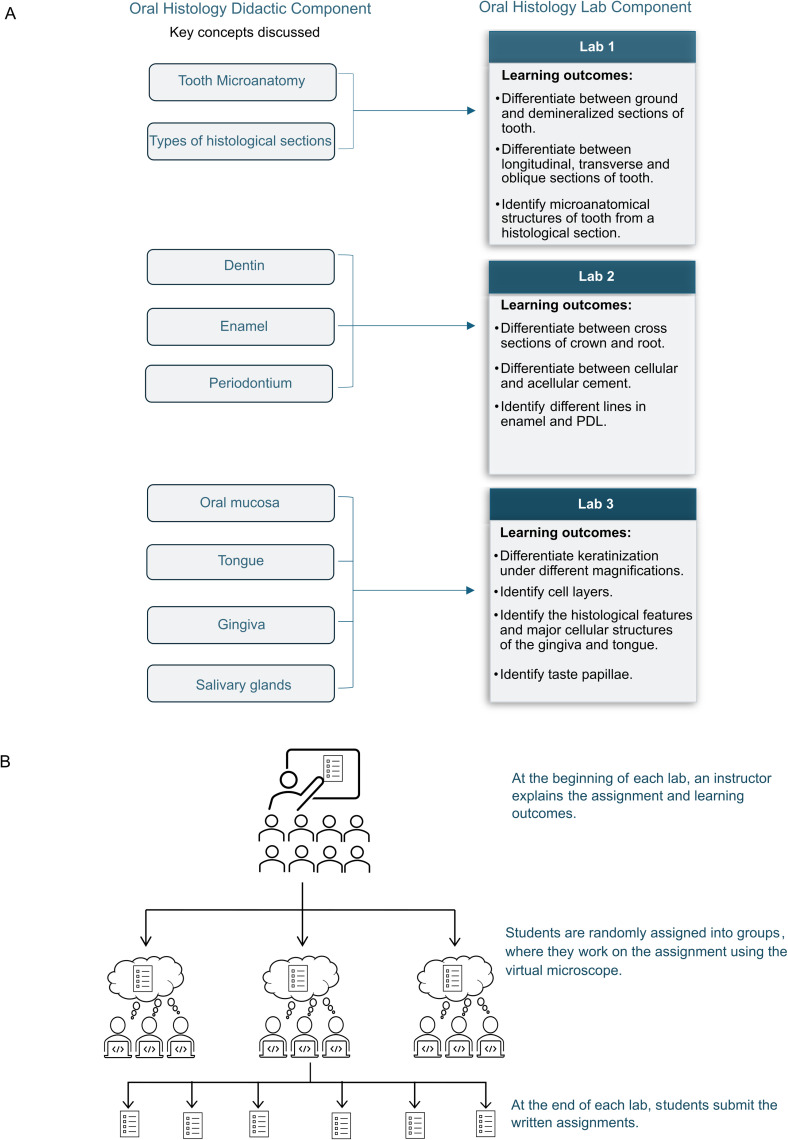
(A) Coordination between the didactic and laboratory components of oral histology in the 1^st^ year Doctor of Dental Surgery (DDS) program. (B) Outline of activities in an oral histology laboratory, where students use a Virtual Microscope (VM) Histoscope. At the beginning of the lab, the instructor explains the assignment and learning outcomes. Students then work on the assignment in small groups. At the end of the lab session, students submit their completed assignments for evaluation.

Three 2-hour mandatory lab sessions were designed for the 1^st^ year DDS students to reinforce and supplement the concepts of oral histology discussed in the didactic lectures (Fig 1A). Three assignments were created for each lab. The assignments included some activities and critical thinking questions students needed to complete in class while using Histoscope. DDS program traditionally enrolls 32 students. At the beginning of the lab, an instructor explained the assignment and the learning outcomes to the students. Students were then divided into groups of 3–4 to work on the assignment. The instructor visits each group during the lab to follow the progress, answer questions, and stimulate discussions. At the end of the session, students submit their completed assignments for evaluation (Fig 1B).

### Study design and population

A convergent, parallel mixed-method study design was chosen to answer our research question. In this type of study design, quantitative and qualitative data are concurrently collected and analyzed [13]. The University of Alberta Research Ethics Board approved this study (ID Pro00104255). All 32 1^st^ year DDS students (DDS-I) who participated in the mandatory lab activities in the Fall 2024 semester were enrolled in the study. The labs were conducted on September 5, September 11 and October 9, 2024. Both the qualitative and quantitative data were anonymized before analysis, and the waiver of participant consent was approved by the ethics committee.

### Data collection

Quantitative data were collected from students’ performance in summative course exams. Five questions on the exam were aimed to evaluate students’ ability to apply their knowledge and understanding of magnification and orientation. The summative exams were in-person, invigilated, closed-book, and students did not have access to Histoscope during the exams. Anonymized difficulty indices for the question were downloaded from the exam platform. The difficulty index for a question represents the percentage of the students who answered that question correctly [14]. The higher the difficulty index, the better the overall class performance on that question.

Qualitative data were collected from students’ written responses to reflective questions. In the last lab assignment, in addition to the questions in the assignment, students were asked to provide written responses to the following reflective questions:

i. Have you improved your understanding of magnification (for histological section) using this virtual microscope?
ii. If yes, how do you think Histoscope has helped you improve your understanding of magnification? If ‘No,’ how can this virtual microscope be improved?
iii. Have you improved your sense of orientation (for the histological section) using this virtual microscope?
iv. If yes, how do you think Histoscope has helped you improve your sense of orientation? If ‘No,’ how can this virtual microscope be improved?

### Data analysis

All data was anonymized before sharing with the research team for analysis. Microsoft Excel was used to calculate descriptive statistics (e.g., percentages) in quantitative data. Manifest content analysis was used to analyze students’ responses to the reflective questions. Manifest content analysis is a multi-step process for summarizing perceptions using categories and subcategories [15].

Text responses to the reflective questions were anonymized and imported into Microsoft Excel. The authors read the data repeatedly and independently before analysis to obtain an overview of the responses. ‘Meaning units’ were identified and independently coded manually by authors. Codes were grouped into subcategories and categories, which the authors established upon discussion. The categories and subcategories were evaluated to confirm that they accounted for all the relevant data.

## Results

### Impact of virtual microscope on dental students’ understanding of the magnification and orientation of a tissue section

Students’ performance (n = 32) on magnification and orientation-related questions was evaluated in summative assessments. All students accurately answered 3 out of 5 questions (Difficulty index = 100%) that required them to apply the knowledge of magnification and orientation. For the other two questions, the difficulty indices were 94% and 91%, meaning that 30 and 29 students were able to answer those questions correctly (Table 1).

**Table 1:** Dental students’ performance on magnification and orientation-related questions in summative assessments, measured using difficulty index.

	Content area	Description of the question	Question type	Required knowledge/understanding	Student performance in the summative exam (Measured from difficulty index)
Q1	Tooth microanatomy	Students were asked to identify striae of Retzius from a given micrograph.	Image labeling from a list of given labels	Magnification Orientation	100%
Q1	Tooth microanatomy	Students were asked to identify lamella from a micrograph.	Image labeling from a list of given labels	Magnification Orientation	100%
Q3	Tooth microanatomy	Students were asked to identify tooth microanatomical structures (rods, interrods, enamel crystallites) and the angle of the section (longitudinal section vs cross-section) from a given electron micrograph.	Multiple choice	Scale of magnification Orientation	100%
Q4	Tooth microanatomy	Students were asked to differentiate between striae of Retzius, cross-striations, Hunter–Schreger bands, and lamellae from a given micrograph.	Multiple choice	Magnification Orientation	94%
Q5	Oral mucosa: Keratinization, salivary glands	Students were asked to identify specific histological sections and compare them with a given oral structure.	Multiple choice	Magnification	91%

### Dental students’ perceptions of using a virtual microscope to comprehend the magnification and orientation of a tissue section

All (100%) students (n = 32) completed and submitted the reflective assignments after the oral histology lab session. In response to questions (i) and (iii), 97% of the class (n = 31) agreed that they had improved their understanding of magnification and orientation (for histological sections) after using the virtual microscope.

**Fig 2:**
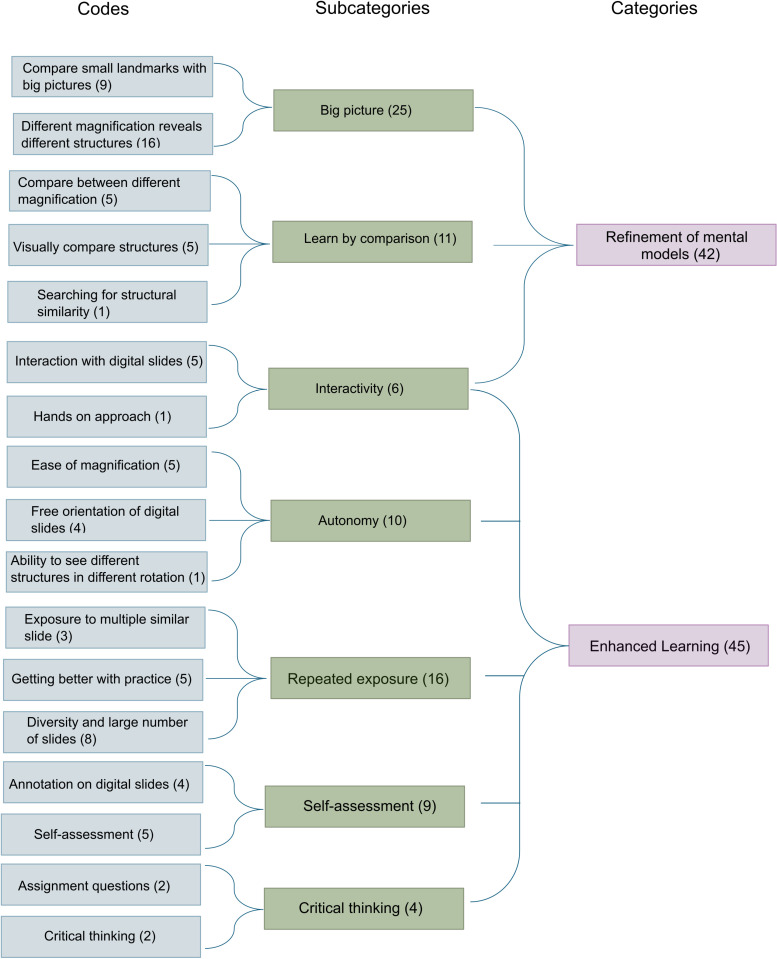
Outline of qualitative data analysis. 17 codes were compiled into 7 subcategories, which were further condensed into 2 categories.

All (100%) students (n = 32) completed questions (ii) and (iv), generating a total of 64 meaning units, each containing single or multiple codes. The analysis of the two prompt questions (ii and iv) was merged. A total of 17 codes were compiled into seven subcategories, which were further condensed into two categories: refinement of mental models and enhanced learning (Fig 2).

### Refinement of mental models

Forty-two meaning units were included in this category, describing strategies adopted by the students to develop and refine mental models while using a VM. 25 meaning units described how students magnified and rotated digital slides, compared a minor feature within the tissue section in the context of the whole slide to build their mental models. Many used the strategy of observing a slide at a low magnification (zoomed out) to understand the entire picture before zooming into specific structural details.

“*It helped me to appreciate the concept of how when something is very zoomed in you can lose sight of what it is actually showing you/ what you are actually looking at. It is helpful to zoom in and out to appreciate the “bigger picture” and then zoom in to a specific section to see the specific details*.” ***(Participant 24)***

A large number of students built their mental model simply by comparing slides at different rotations and magnifications, resulting in the inclusion of 11 meaning units under the subcategory of learning by comparison. According to this group of students, viewing tissue sections at different magnifications and orientations helped them comprehend how a particular cellular structure is situated in the surrounding environment. Students also attributed the interactive nature of Histoscope, which included the ability to magnify and rotate a digital slide seamlessly, to help build their mental model of the tissue section.

*“It helped me look for key landmarks, and use those when identifying the object and thus orientating myself to what I am looking at.” (sic)* **(*Participant 24*)**

*“It has helped me understand how to identify the structures in different orientations*.” **(*Participant 25*)**

### Enhanced learning

Forty-five meaning units were included in the category of enhanced learning. This category includes the features of the virtual microscope and the lab assignments that helped students improve their learning experiences, including the understanding of magnification and orientation. The subcategories include autonomy, repeated exposure, self-assessment, critical thinking, and interactivity. Many students ascribed their autonomy and seamless interaction with digital slides to their improved understanding of magnification and orientation, resulting in 10 meaning units. Compared to a traditional light microscope, students preferred observing the real-time changes in the viewing of digital slides while changing the magnification and orientation with a mouse click.

*“Typical microscopy requires massive jumps in magnification that can obscure how we understand the progression in these scales, but histoscopes digital allows us to understand the transition and context of increased magnification.”* **(*Participant 19*)**

Students appreciated the repeated exposure to a large and diverse collection of digital slides through the VM, resulting in 16 meaning units in this subcategory. Many mentioned that exposure to diverse digital slides improved their familiarity with tissue sections in different orientations. Others consider the VM to be a tool for self-assessing learning, generating 10 meaning units. Some students mentioned that the assignment questions promoted critical thinking and improved their knowledge of magnification and orientation (4 meaning units). (Table 2).

**Table 2:** Categories, subcategories, and representative quotes.

Categories	Subcategories	Representative quotes
Refinement of mental models (42 meaning units)	Big picture (25 meaning units)	It helped me visualize the structures such as the cells and tissue in more detail which I did not get from a single image. I am now able to understand the different layers of information you receive from different types of magnification where lower magnification can help you see the structure in its entirety but higher magnification allows me to see the individual cellular structures in more detail. ***(Participant 11)*** I think Histoscope helped put the smaller histological sections into perspective when comparing and looking at the overall structure of the tooth. It helps you understand smaller features and landmarks and compare relative to the big picture by being able to zoom in and out of the diagram. ***(Participant 4)*** By zooming in an out of the image, I was able to see the magnified slides in a bigger context and understand the whole picture before zooming into the individual layers. ***(Participant 22)***
	Learning by comparison (11 meaning units)	It helps to see the tissues under different magnifications and compare the structures that can be viewed at different magnifications. It gives me a better idea of how the sizes of different structures compare to one another. ***(Participant 16)*** It has allowed me to see the microscopic structures talked about in our lectures. While drawn diagrams can be helpful, it is better to see the structures in their context. By being able to magnify these structures, I can see everything else around it and how it interacts with other structures in its environment. ***(Participant 15)***
	interactivity (6 meaning units)	The interactive natrue has helped my understanding of gingiva and mucosa. (***Participant 10)*** Similarily, being able to start at a low magnification as well as the title of slide stating what I am looking at, I am able to orient what structure/tissue/cells I am looking at. Additionally, the labels on histoscope sometimes help me with orienting by pointing to significant structures or areas on the slide. ***(Participant 23)***
Enhanced Learning (45)	Autonomy (10 meaning units)	Histoscope has improved my understanding of magnification through its ability to zoom and seamlessly transition between levels of magnification that can depict entire tissues or small sections with individual cells visible. Typical microscopy requires massive jumps in magnification that can obscure how we understand the progression in these scales, but histoscopes digital allows us to understand the transition and context of increased magnification. ***(Participant 19)*** Histoscope has helped me improve my sense of orientation by allowing me to continuously flip the image in the histoscope program. This allows me to understand how different orientations can present themselves. This has allowed me to understand that a lower magnification level upon initial view can help orient myself correctly based on macroscopic structure view. ***(Participant 2)***
	Repeated exposure (16 meaning units)	With practice looking at a variety of slides, I am becoming more familiar with how they should be oriented when I identify some of the key structures on them. ***(Participant 7)*** Histoscope has helped me improve my sense of orientation by allowing me to continuously flip the image in the histoscope program. This allows me to understand how different orientations can present themselves. This has allowed me to understand that a lower magnification level upon initial view can help orient myself correctly based on macroscopic structure view. ***(Participant 4)*** Yes, my sense of orientation has improved as I was able to have more practice with Identifying different slides. ***(Participant 32)***
	Self-assessment (9 meaning units)	Also, I really like the label function so that I can check my knowledge and use it as a resource to study for exams. ***(Participant 30)*** It is helpful to see different preparations and test myself with identifying structures. ***(Participant 1)***
	Critical thinking (4 meaning units)	This was a hands on approach where I am able to think critically with more real life slide preparations. I found this useful given that not all slide preparations are straight forward and you really need to understand the material to complete your work. (***Participant*** 32)
	interactivity (6 meaning units)	By having interactive labeling on Histoscope, I can see where the structures are located and allow myself to orient where I am looking at based on the structure. ***(Participant 15)*** It has helped me identify tooth and gingiva structures much better. The interactive nature of histoscope and the assignments have improved my understanding. ***(Participant 28)***

*“Let’s me see different preps in various orientations which gives me greater ability to orient myself.”* ***(Participant 17)***

*“It is useful to use Histoscope because it lets me view new microscopic images of teeth and other components of the oral cavity giving me a chance to practice using a lot of the new labels we’ve learned in lecture. It has shown me the diversity that a lot of these structures can come in because no two preparations will look exactly the same.”* ***(Participant 30)***

Histoscope offers interactive annotations. Users can reveal or hide the annotation of a digital slide. This interactive nature of the VM was recognized to improve the compression of magnification and orientation. Several students mentioned using the interactive labeling of Histoscope to identify the location of a structure and self-assess their sense of orientation.

*“By having interactive labeling on Histoscope, I can see where the structures are located and allow myself to orient where I am looking at based on the structure.”* ***(Participant 15)***

## Discussion

Spatial abilities are critical for the oral health professions, with their importance highlighted in the fact that entrance exams to dental professional programs include a component to test these perceptual abilities [16,17]. Spatial abilities are also critical for forming 3D mental models that help students understand the spatial relationships of anatomical and histological structures. VMs are gradually becoming popular as a teaching tool. However, a lack of hands-on experience with traditional light microscopes and glass slides is still a concern for many educators. Though many groups have reported that there is an improvement or no difference in knowledge and/or performance of students using VM vs optical microscopes [18,19], reports on the impact of VMs on students’ understanding of magnification and orientation are scarce. Our study explored dental students’ measured and perceived knowledge of magnification and orientation after using VMs in an oral histology lab. Our research revealed that dental students perceived improvement in their knowledge of magnification and orientation after using VMs and demonstrated an adequate understanding of magnification and orientation in summative assessments. Qualitative analysis of student reflection data illustrated how VMs can be used to help develop 3D mental models of the tissue structure. Studies have shown that though there are sex differences in spatial ability, these differences are less impactful in smaller scales like histological images (vs gross anatomy) and can be reduced with practice [20], which VMs afford.

The Mike Petryk School of Dentistry at the University of Alberta has gone through a curriculum renewal, shifting the DDS program from a traditional format, consisting of a large number of smaller siloed disciplinary courses, to a format including modular courses. In this new format, foundational science is taught along with patient care, operative, and occlusion to foster vertical and horizontal integration of knowledge. However, this format limits the number of questions that can be asked from each discipline in one summative exam. The summative exam of DDS-I included multiple questions evaluating students’ knowledge of oral histology and tooth microanatomy. However, five questions were aimed to challenge students with their understanding of tissue orientation and magnification.

Histoscope, the VM used in this study, archives digital slides that were scanned using a Z-stacking technology, enabling users to effortlessly magnify any section of the tissue to visualize structural details in high resolution [12,21]. Users can also rotate a digital slide and hide and reveal its annotations by clicking a button. This interactive feature of Histoscope appeared to play a key role in many other aspects, and thus, the subcategory ‘interactivity’ was included in both categories: Refinement of mental models and enhanced learning. The interactive magnification and rotation helped students compare structures and build mental models, and some groups have hypothesized that the perspectival appearance changes during the rotation of an object impact a student’s ability to mentally rotate objects [22]. While we were initially concerned that the navigation of the VM technology might increase the extrinsic load experienced by students, the explicit demonstration of orientation and magnification by the VM may reduce the burden of having to rely exclusively on the student’s intrinsic ability to rotate objects mentally. Many students used the interactive labeling feature of the VM as a guide to locate specific structures. Others use the same interactive labeling to self-assess their learning of oral histology.

Traditional light microscopy lets students touch and interact with the glass slides directly. VMs have compensated for this feature by offering the experience of manipulating and observing a slide rotate and magnify in real-time. The ability to spatially zoom in to microscopic structures using a VM allows students to simultaneously appreciate the details of the small-scale structures and the larger context within which the structures are situated. The readily accessible diverse slide collection and interactive annotation are the other two features of VMs essential for improving students’ learning experience. To enhance students’ learning experience with VMs, well-designed lab sessions and critical thinking questions are also essential. Although only 4 meaning units recognized the impact of guiding questions and assignments, it is key to stimulate collaborative, engaging, and interactive lab sessions with VMs.

### Limitations

We acknowledge several limitations of our study. The findings of this study are based on a small sample size from a single cohort of dental students, which limits the generalizability of the findings. Control groups are absent. Demographic data from the study participants and faculty perceptions were not collected. A small number of questions from a limited array of tissue sections were used to assess students’ understanding of magnification and orientation. Further studies with an extended assessment tool are needed to better analyze students’ knowledge and comprehension of tissue magnification. This study relies heavily on students’ self-reported qualitative data. Besides many known benefits of self-reporting, we acknowledge that self-reported data is often affected by factors like the responder’s introspective ability and capacity to interpret questions [23]. Additionally, the use of exemplar images in the VM does not allow students to appreciate variations in structure present in real-life specimens.

## Conclusion

The knowledge of microanatomy is an essential cornerstone for dental, medical, and many other health professional education. Although our study has multiple limitations, it shows the positive impacts perceived by the dental students of using a VM to study oral histology and sheds light on the strategies employed by dental students to improve their senses of magnification and orientation while using this teaching tool.Future studies can evaluate the long-term impact of a VM on students’ academic achievements, engagement, and knowledge retention.

## References

[1] Mazzarini M, Falchi M, Bani D, Migliaccio AR. Evolution and new frontiers of histology in bio-medical research. Microsc Res Tech. 2021;84(2):217–37. doi: 10.1002/jemt.23579 32915487 PMC8103384

[2] Hamilton PW, Wang Y, McCullough SJ. Virtual microscopy and digital pathology in training and education. APMIS. 2012;120(4):305–15. doi: 10.1111/j.1600-0463.2011.02869.x 22429213

[3] Sander B, Golas MM. HistoViewer: an interactive e-learning platform facilitating group and peer group learning. Anat Sci Educ. 2013;6(3):182–90. doi: 10.1002/ase.1336 23184574

[4] Virtual Microscopy. [Website]. Available from: https://www.virtual-microscopy.net/. [Last accessed on October 4, 2024].

[5] Triola MM, Holloway WJ. Enhanced virtual microscopy for collaborative education. BMC Med Educ. 2011;11:4. doi: 10.1186/1472-6920-11-4 21269474 PMC3037351

[6] Sharmin N, Chow A, Dong A. A comparison between virtual and conventional microscopes in health science education. CJLT. 2023;49(2):1–20. doi: 10.21432/cjlt28270

[7] Rosai J. Why microscopy will remain a cornerstone of surgical pathology. Lab Invest. 2007;87(5):403–8. doi: 10.1038/labinvest.3700551 17401434

[8] Hortsch M, Koney NK, Oommen AM, Yohannan DG, Li Y, de Melo Leite AC, et al. Virtual microscopy goes global: the images are virtual and the problems are real. In: Biomedical Visualisation: Volume 16‒Digital Visualisation in Biomedical Education. Cham: Springer International Publishing; 2023. p. 79-124. 10.1007/978-3-031-30379-1_5 37524985

[9] Xu CJ. Is virtual microscopy really better for histology teaching? Anat Sci Educ. 2013;6(2):138. doi: 10.1002/ase.1337 23184604

[10] DeRose JA, Doppler M. Guidelines for understanding magnification in the modern digital microscope era. Micros Today. 2018;26(4):20–33. doi: 10.1017/s1551929518000688

[11] Jones MG, Andre T, Superfine R, Taylor R. Learning at the nanoscale: The impact of students’ use of remote microscopy on concepts of viruses, scale, and microscopy. J Res Sci Teach. 2003;40(3):303–22. doi: 10.1002/tea.10078

[12] Sharmin N, Chow AK, Dong AS, Milos NC. Histoscope: a web-based microscopy tool for oral histology education. Healthc Inform Res. 2021;27(2):146–52. doi: 10.4258/hir.2021.27.2.146 34015880 PMC8137871

[13] Creswell JW, Plano Clark VL. Designing and Conducting Mixed Methods Research. 2nd ed. Los Angeles, CA: SAGE Publications; 2011.

[14] Iñarrairaegui M, Fernández-Ros N, Lucena F, Landecho MF, García N, Quiroga J, et al. Evaluation of the quality of multiple-choice questions according to the students’ academic level. BMC Med Educ. 2022;22(1):779. doi: 10.1186/s12909-022-03844-3 36369070 PMC9652897

[15] Elo S, Kyngäs H. The qualitative content analysis process. J Adv Nurs. 2008;62(1):107–15. doi: 10.1111/j.1365-2648.2007.04569.x 18352969

[16] ADA. Dental Admission Test (DAT®). [Website]. Available from: https://www.ada.org/education/testing/exams/dental-admission-test-dat. [Last accessed on December 4, 2024].

[17] Canadian Dental Association. Dental Aptitude Test. [Website]. Available from: https://www.cda-adc.ca/en/becoming/dat/. [Last accessed on December 4, 2024].

[18] Kuo K-H, Leo JM. Optical versus virtual microscope for medical education: a systematic review. Anat Sci Educ. 2019;12(6):678–85. doi: 10.1002/ase.1844 30414261

[19] Mione S, Valcke M, Cornelissen M. Evaluation of virtual microscopy in medical histology teaching. Anat Sci Educ. 2013;6(5):307–15. doi: 10.1002/ase.1353 23463716

[20] Yuan L, Kong F, Luo Y, Zeng S, Lan J, You X. Gender differences in large-scale and small-scale spatial ability: a systematic review based on behavioral and neuroimaging research. Front Behav Neurosci. 2019;13:128. doi: 10.3389/fnbeh.2019.00128 31275121 PMC6591491

[21] Ray S. Applied photographic optics. Routledge; 2002.

[22] Stewart EEM, Hartmann FT, Morgenstern Y, Storrs KR, Maiello G, Fleming RW. Mental object rotation based on two-dimensional visual representations. Curr Biol. 2022;32(21):R1224–5. doi: 10.1016/j.cub.2022.09.036 36347228

[23] Paulhus D L, Vazire S. The self-report method. Handbook of research methods in personality psychology. 2007; 1(2007):224-39.

